# Associations Between Ambient PM_2.5_ and Thyroid Hormones in Pregnant Persons in Puerto Rico

**DOI:** 10.3390/toxics13010058

**Published:** 2025-01-15

**Authors:** Trenton Honda, Trenton D. Henry, Laura Corlin, Kipruto Kirwa, Akram Alshawabkeh, Julia R. Varshavsky, Winston Kennedy, José F. Cordero, Carmen M. Velez Vega, Zaira Y. Rosario Pabon, John D. Meeker, Helen Suh

**Affiliations:** 1 School of Clinical and Rehabilitation Sciences, Northeastern University, Boston, MA 02115, USA; 2 Department of Public Health and Community Medicine, Tufts University School of Medicine, Boston, MA 02111, USA; 3 Department of Civil and Environmental Engineering, Tufts University School of Engineering, Medford, MA 02155, USA; 4 Department of Environmental Health, Boston University School of Public Health, Boston, MA 02118, USA; 5 Department of Civil & Environmental Engineering, Northeastern University, Boston, MA 02115, USA; 6 Department of Public Health and Health Sciences, Northeastern University, Boston, MA 02115, USA; 7 Department of Epidemiology and Biostatistics, University of Georgia, Athens, GA 30602, USA; 8 Department of Social Sciences, UPR Medical Sciences Campus, University of Puerto Rico Graduate School of Public Health, San Juan, PR 00936, USA; 9 Department of Environmental Health Sciences, University of Michigan School of Public Health, Ann Arbor, MI 48109, USA

**Keywords:** air pollution, PM_2.5_, thyroid hormones, maternal health

## Abstract

Introduction: This study investigates associations between fine particulate air pollution (PM_2.5_) exposure and thyroid hormone levels during pregnancy in Puerto Rican individuals, a vulnerable population facing socioeconomic and environmental disparities. Methods: This research draws on data from the PROTECT cohort study and involves 1040 participants to measure the effect of PM_2.5_ on developmentally important thyroid hormones (TSH, T3, T4, and FT4). Pollution concentrations were linked to participant locations using EPA air quality data and analyzed across two visits during gestational weeks 16–20 and 24–28. Results: The results suggest that PM_2.5_ exposure is positively associated with maternal T3, T4, and FT4 levels but not TSH. These effects vary by timing, with T3 showing stronger associations later in pregnancy and T4/FT4 earlier. Nonlinear dose–response relationships were observed, suggesting thresholds for certain hormones. Discussion: These findings support previous studies linking altered thyroid hormones to adverse birth outcomes and highlight the potential role of air pollution in disrupting maternal thyroid function and its implications for fetal development, calling for further research into mechanisms and interventions to mitigate these risks.

## 1. Introduction

Early in gestational development, a human fetus is highly dependent on maternal thyroid hormones for the regulation of fetal physical growth [1], placental development [2], and brain maturation [3]. In fact, the fetus does not develop its own thyroid hormones until the second trimester. Several critical brain function milestones occur throughout the second and third trimesters as the neonate’s own thyroid hormone production begins.

Maternal thyroid regulation may have a particularly important impact on neonatal birth and cognitive outcomes. Both hypo- and hyperthyroidism are dangerous in pregnancy: the dysregulation of maternal or fetal thyroid levels can result in adverse pregnancy and neurodevelopmental outcomes including low birth weight, miscarriage, and cognitive impairment [1,4,5]. Clinical interventions, such as iodine supplementation or the administration of antithyroid agents, are sometimes required to ensure healthy pregnancy [6].

Air pollution, most notably ambient fine particulate matter ≤ 2.5 μm in diameter (PM_2.5_), may affect maternal thyroid regulation [7]. Through this pathway, PM_2.5_ may lead to adverse birth outcomes, including fetal developmental pathologies, low birth weight, preterm birth, and infant and neonatal mortality [8,9,10,11,12,13,14]. Furthermore, research has implicated air pollution in disrupted neurodevelopment, which may lead to delayed global, verbal, and psychomotor development during infancy. While poorly understood, it is possible that PM_2.5_-associated perturbations in thyroid hormone levels, such as thyroxine (T4), may lead to such adverse neurodevelopmental outcomes [15,16,17,18,19]. Howe et al., for example, observed that increased exposure to PM_2.5_ was associated with higher total T4, although other studies have reported inverse associations.

Moreover, there are racial and ethnic inequities in PM_2.5_ exposure that may contribute to an increased risk of adverse neurodevelopmental outcomes among historically marginalized and vulnerable communities that are burdened by multiple environmental and social stressors [20,21,22,23]. It is, therefore, important to understand the role that air pollutants play in disrupting the thyroid hormone regulation of prenatal brain development, especially in vulnerable populations.

The inhabitants of the USA island territory of Puerto Rico, who are primarily Hispanic, are an example of such a vulnerable population. Puerto Rican health is impacted by structural racism and discrimination [24,25,26], as well as economic disadvantage, with a poverty rate of 41.7%, and a median household income of just USD 21,967, compared to mainland USA’s USD 69,021 [27,28]. In addition, Puerto Rico is burdened with 18 Superfund sites, which has resulted in disproportionately high exposure to chemicals such as phthalates, heavy metals, and polycyclic aromatic hydrocarbons [29,30,31], the adverse effects of which may be enhanced by social stress and climate vulnerability [29,32,33]. The health impacts of these social and environmental factors are particularly detrimental to maternal and fetal health, resulting in low birth weight and preterm birth rates consistently exceeding those seen on the mainland [34], maternal mortality that is higher than in other Hispanic populations [35], and elevated infant mortality levels compared to non-Hispanic whites [36]. Despite these inequities and known detrimental environmental exposures, few studies have examined the degree to which air pollutants may contribute to these adverse maternal and fetal outcomes, and none have explored how these exposures may perturb normal maternal thyroid physiology [37].

To address these gaps in the scientific literature, we sought to determine whether exposure to air pollution is associated with differences in thyroid hormone levels during a critical period of fetal development among a susceptible and understudied population of pregnant Puerto Rican individuals.

## 2. Methods

### 2.1. Study Design and Population

This study draws from 1040 pregnant persons enrolled in the ongoing Puerto Rico Test site for Exploring Contamination Threats (PROTECT) prospective cohort study, which investigates environmental impacts on birth outcomes among Puerto Rican birth parents. These participants were recruited from the northern coast of the island, which has a high pollution burden. Participants were eligible if they (1) were 18–40 years old, (2) had no pre-existing medical or obstetric conditions that would complicate pregnancy, such as diabetes, (3) did not become pregnant via in vitro fertilization, and (4) refrained from oral contraceptives three months before pregnancy [38]. Between 2011 and 2019, we measured blood levels of thyroid hormones [thyroid-stimulating hormone (TSH), Triiodothyronine (T3), thyroxine (T4), and free T4 (FT4/FT4_2)] among pregnant PROTECT participants. During the study, birth parents were involved in up to two prenatal clinic visits (18 and 26 ± 2 weeks of gestation) and one in-home visit at approximately 22 weeks (±2 weeks) of gestation. In each visit, participant behavioral and demographic information, pregnancy status, and spot urine and/or blood samples were collected. Participant follow-up continued through pregnancy and delivery. Data on birth outcomes and neonatal status were also collected. Further details of the study’s design have been described previously [38,39].

### 2.2. Institutional Review Board

The institutional review boards at the University of Puerto Rico, Northeastern University (Approval Number IRB #21-10-18), University of Michigan, and the University of Georgia approved the PROTECT study.

### 2.3. Exposures

**Figure 1:**
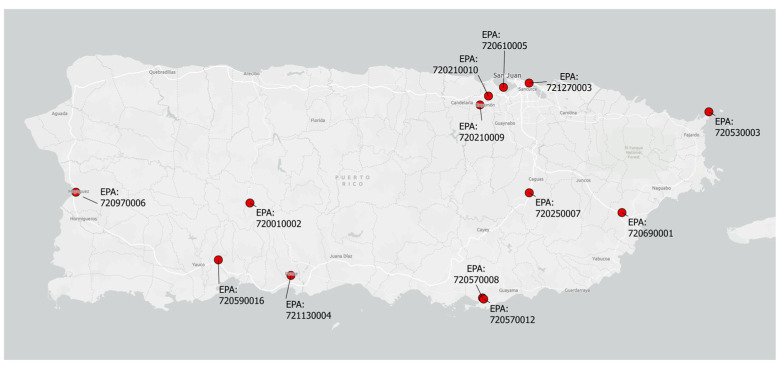
Map of Puerto Rico and EPA monitoring sites.

As in our previous studies in this cohort, the exposure of interest was PM_2.5_, which was measured daily or semiweekly at 13 stationary ambient monitoring sites of the USA’s Environmental Protection Agency (EPA) air quality system (AQS) and retrieved from the AQS data mart (Figure 1) [40]. In total, 3-, 7-, and 30-day moving average exposures preceding each participant’s study visits were estimated and linked to each participant using the centroid of each participant’s residential municipality using inverse distance weighting (squared) [41].

### 2.4. Outcomes

The primary outcomes of interest were serum thyroid hormone concentrations: TSH (μIU/mL), T3 (ng/mL), T4 (μg/dL), and FT4_2 (ng/dL). Hormone levels were measured in blood samples collected at study visits one and three in-clinic (at gestational weeks 16–20 and 24–28, respectively). Blood samples were gathered in whole-blood collection tubes, frozen at −80 °C, and shipped in dry-ice containers to University of Michigan (Ann Arbor, MI, USA) for analysis. Analyte levels were measured using immunoassay at the University of Michigan School of Public Health Epidemiology Department’s Central Ligand Assay Satellite Services (CLASS) laboratory and are described in more detail elsewhere [42]. Concentrations of all hormones were log-transformed to facilitate linear modelling and to aid interpretation [43]. For all outcomes, analyte values that fell below the limit of detection (LOD) were assigned the value LOD/2^½^ [44].

### 2.5. Covariates

Covariates and categorical cut points were selected *a priori* based on a previous analysis of this cohort [12]. They included demographic information [age (continuous), race (categorical, self-reported by participants as White, Mestiza, Black, Multi-racial, or Other)], indicators of socioeconomic status [employment status (categorical), household income (categorical), education (categorical)], medical history and health behaviors [parity (categorical), previous adverse pregnancy outcomes (binary), exercise (categorical), BMI (categorical), alcohol consumption (categorical), smoking status (categorical)], marital status (categorical), municipality (categorical), visit month (categorical), gestational age (continuous), and batch (date) of laboratory hormone processing (categorical). Covariate missingness is noted in Supplemental Table S1 and Figure S1.

### 2.6. Statistical Analysis

#### 2.6.1. Models

We used linear mixed effects models with random intercepts for each participant to determine associations between ambient PM_2.5_ and log-transformed TSH, T3, T4, and FT4 across both pregnancy visits [43]. Fully adjusted models included age, education, marital status, parity, history of adverse pregnancy outcome, employment status, household income, race, exercise, BMI, alcohol consumption, smoking status, municipality, visit month, gestational age, and date of laboratory analysis. Cross-sectional linear models were also used to examine associations by visit. We exponentiated coefficients to yield estimates of association in percent difference per 10 μg/m^3^ in exposure. We further examined dose–response and nonlinear effects using generalized additive mixed models (GAMMs) with cubic splines.

#### 2.6.2. Missing Data and Imputation

We used multiple imputation with chained equations to create 30 imputed data sets with non-missing covariates [45]. PM_2.5_ exposure and hormone values were not imputed. Regression was performed on all 30 data sets, after which results were pooled to obtain final effect estimates [46]. Estimates were subsequently exponentiated and converted to percentages.

#### 2.6.3. Software

All analyses were performed using R Statistical Software version 4.2.2 with packages MICE, lme4, merTools, and mgcv [45,46,47].

## 3. Results

Table 1 shows the demographic details of the participants. Our analyses included 1040 pregnant persons whose average age was 26.9 ± 5.5 years. For 43% of the participants, this was their first pregnancy, and 53% were married when enrolled in the study.

During the study period (2011–2020), the mean island-wide PM_2.5_ concentration was 11.9 ± 12.2 μg/m^3^ (median 8.2, IQR: 10.7), and mean participant exposures for 3-, 7-, and 30-day moving averages were 8.0 ± 5.9, 8.2 ± 5.3, and 8.1 ± 4.4 μg/m^3^, respectively.

“Visits 1 & 3” are cross-sectional multiple regression analyses; “all” includes measures from both visits with random intercepts at the level of the participant.

Risk estimates are expressed per 10 μg/m^3^ difference in estimated PM_2.5_ concentrations.

Fully adjusted models are adjusted for age, race, employment, family income, education, parity, prior negative pregnancy outcomes, exercise, BMI, alcohol use, smoking exposure, marital status, municipality of residence, seasonality (month), gestational age, and batch (date) of laboratory hormone processing.

**Figure 2:**
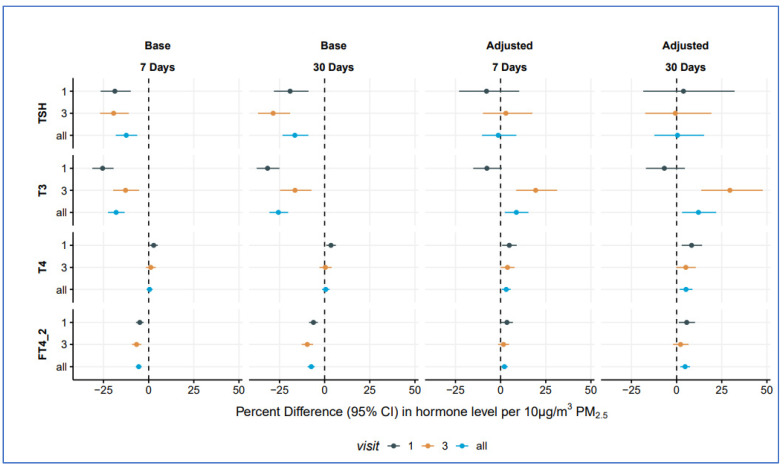
Forest plot of associations between PM_2.5_ and maternal thyroid hormones by visit and exposure length.

Figure 2 shows associations between PM_2.5_ exposure and thyroid hormone levels at visits one (gestational weeks 16–20), three (gestational weeks 24–28), and across the full pregnancy for 7-day and 30-day moving average PM_2.5_ exposure.

*Visit 1 cross-sectional analyses*: For TSH and T3, we observed consistent inverse associations in our cross-sectional base models at Visit 1 in both 7-day and 30-day moving average exposures. In fully adjusted models, associations for both TSH (7-day: %-difference −7.8, 95% CI: −23.0, 10.3; 30-day: %-difference 3.8, 95% CI: −18.5, 32.1) and T3 (7-day: %-difference −7.6, 95% CI: −15.2, 0.7; 30-day: %-difference −6.8, 95% CI: −17.0, 4.6) were non-significant. For T4, statistically significant and positive associations were observed in base models, with larger magnitude associations observed in fully adjusted models, where a 10 μg/m^3^ increase in 7-day moving average PM_2.5_ exposure was associated with a 4.8% (95% CI: 0.8, 8.9) increase, and a 10 μg/m^3^ increase in 30-day moving average PM_2.5_ exposure was associated with an 8.3% (95% CI: 2.8, 14.1) increase. Similar results were observed for FT4 in adjusted models, where a 10 μg/m^3^ increase in 7-day and 30-day average PM_2.5_ exposure was associated with a 3.5% (95% CI: 0.2, 6.8) and a 5.6% (95% CI: 1.2, 10.2) increase in FT4, respectively.

*Visit 3 cross-sectional analyses*: As in our Visit 1 analyses, we observed consistent inverse associations in our cross-sectional analyses at Visit 3 for 7- and 30-day moving average exposures for base models for TSH and T3. When fully adjusted, TSH estimates were non-significant, while, for T3, we observed significant and positive associations for both 7-day (%-difference: 19.4, 95% CI: 8.6, 31.3) and 30-day (%-difference: 29.5%, 95% CI: 13.6, 47.7) exposure windows. Effect estimates for T4 and FT4 were non-significant in fully adjusted models.

*Mixed effects analysis over all visits*: When examining associations across both visits, with random intercepts for each participant in fully adjusted models, we observed significant and positive associations with T3 (7-day: %-difference 8.7, 95% CI: 2.3, 15.4; 30-day: %-difference 12.1, 95% CI: 3.0, 21.9), T4 (7-day: %-difference 3.1, 95% CI: 0.7, 5.6; 30-day: %-difference 5.2, 95% CI: 1.7, 8.8), and FT4 (7-day: %-difference 2.1, 95% CI: 0.4, 3.9; 30-day: %-difference 4.7, 95% CI: 2.1, 7.4), while associations were null for TSH.

**Figure 3:**
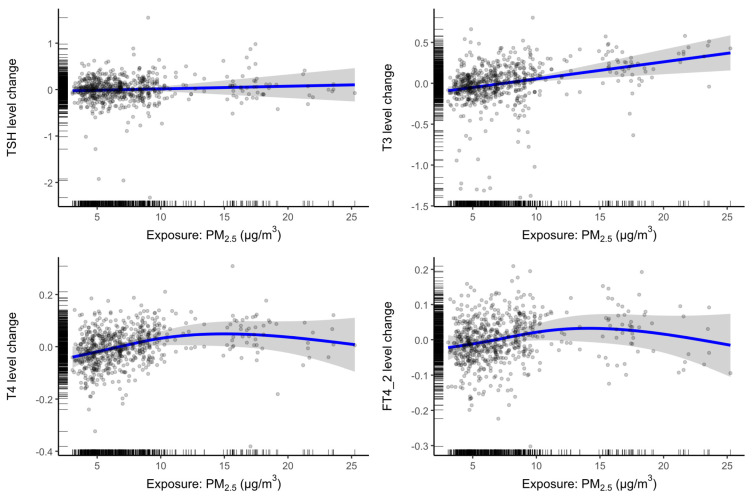
Cubic spline GAMMs of hormone dose responses for all visits at 30-day exposure. Circles are observed thyroid hormone levels; blue line is the estimated thyroid hormone level given the PM_2.5_ exposure concentration; grey bands are 95% confidence intervals around the estimates.

*Dose response*: To explore nonlinear associations, we used generalized additive mixed models (GAMMs) with cubic splines for the 30-day moving average longitudinal exposures. Figure 3 shows these results. Associations are null for TSH and increase linearly for T3. However, for T4 and FT4, there is the suggestion of a threshold effect, whereby associations increase linearly until approximately 10 μg/m^3^, after which associations become non-significant.

The complete case analysis of 30-day exposure was adjusted with all covariates noted above: age, race, employment, family income, education, parity, prior negative pregnancy outcomes, exercise, BMI, alcohol use, smoking exposure, marital status, municipality of residence, and seasonality (month), gestational age, and batch (date) of laboratory hormone processing.

## 4. Discussion

Our study is the first to examine the association between PM_2.5_ and developmentally important thyroid hormones in a population of pregnant persons in Puerto Rico. We observed that air pollution exposure was associated with increased levels of T3, T4, and FT4, while associations limited to each visit date found some heterogeneity in associations. For example, T3 was not associated with PM_2.5_ in early pregnancy, but it was in later pregnancy. Conversely, T4 and FT4 were associated with air pollutant exposures in early pregnancy, but not later in pregnancy. We also found evidence of nonlinear dose–response relationships for T4 and FT4. No significant associations were observed between PM_2.5_ and TSH.

The prior literature examining the impacts of PM_2.5_ on thyroid hormones is incomplete and inconsistent. For example, several prior studies have examined TSH, FT3, and FT4, but the literature examining associations with T4 was limited to newborns and not pregnant persons [48]. To our knowledge, no previous study has explored the associations between PM_2.5_ and T3. The prior literature examining the impact of PM_2.5_ on TSH is likewise mixed, with several studies observing null associations, similar to our findings [7,15,16,49,50], while other studies identified strong positive or inverse associations [15,51]. Although no prior study has examined T3, reports of associations between PM_2.5_ and FT3 are mixed, with studies observing either null, positive, or inverse associations [15,50,52]. Most of the prior literature on FT4 has identified significant inverse or null associations [7,15]. Potential explanations for this heterogeneity in the prior literature include differences in study design, PM_2.5_ components, and study population and geography.

There are several potential biological mechanisms that may explain the changes in thyroid hormones we observed to be associated with PM_2.5_ exposure. For example, there is some evidence to suggest that PM_2.5_ interrupts the Hypothalamic–Pituitary–Adrenal axis, which regulates both the hypothalamic production of thyrotropin-releasing hormone and the pituitary secretion of TSH [53]. However, the null associations we observe with TSH make such an explanation less likely in our population. In fact, as T3 and T4 are thyroid products, while TSH is a pituitary product, the associations we observe may suggest that PM_2.5_ has a greater impact on the thyroid gland than the pituitary. It is also possible that the impact of other hormones that influence TSH (e.g., cortisol, growth hormone) may obscure the impact of PM_2.5_ on TSH. Additionally, there is the possibility of direct damage to the maternal thyroid gland through either inflammatory processes or particle translocation, which would be consistent with the observed differences we see in thyroid hormones, while associations with TSH are null. Placental damage by fine particulates could also be a contributory cause, as placental beta Human Chorionic Gonadotropin interacts with TSH and thyroid hormone receptors in early pregnancy, and placental injury could disrupt this physiologic process [54,55]. Lastly, because the fetus is dependent upon maternal thyroid hormones in early pregnancy—whereas, in later pregnancy, the fetal thyroid produces its own TSH, T3, and T4—it is possible that different mechanisms and their interactions with maternal thyroid hormone production may be responsible for the findings we see in the Visit 1 and Visit 3 cross-sectional analyses. Further research is needed to better elucidate the potential mechanisms.

Importantly, elevations in maternal thyroid hormones may have lifelong impacts to health. Prior studies have shown that higher levels of maternal thyroid hormones—even within normal limits—are associated with lower birth weight and small-for-gestational-age status of the infant [56], both of which have been associated with decrements in academic achievement and professional attainment in later life [57]. This is consistent with recent animal studies that have shown that exposure to elevated thyroid hormone levels in the neonatal period have been associated with growth and developmental delays, likely due to an abnormal hypothalamic–pituitary axis setpoint [58]. Given that we have observed lower birth weight to be associated with prenatal PM_2.5_ exposure in this cohort, it is possible that the effect of PM on maternal thyroid hormone levels is a pathway by which PM impacts birth weight [13]. Additionally, in our prior work in this cohort, we identified ambient PM components to be associated with perturbations in markers of neurological integrity, which can be impacted by alterations in thyroid hormone levels [59]. Furthermore, in male rat and mouse fetuses, elevated exposure to thyroid hormone has been associated with significant reductions in testicular development and in adult testicular size, likely due to the epigenetic modification of genes resulting in the arrest of Sertoli cell proliferation [58,60], which is consistent with our and others’ prior work showing PM_2.5_ exposure to negatively affect sperm quality metrics [61]. In sum, the detrimental health effects of perturbations in fetal exposure to maternal thyroid hormones may reverberate throughout the lifespan.

Our study has several key strengths. These include a relatively large sample size, use of thyroid hormone measures across pregnancy, and availability of data on several important potential confounders. Despite these strengths, limitations of our study include the use of regional air pollution exposure measurements rather than personal or hyperlocal exposure measurements, which may have introduced exposure misclassification into our study. However, this type of random exposure misclassification would be likely to bias results to the null, rather than resulting in systematic error away from the null. In addition, we were not able to assess the potential impact of occupational or non-occupational endocrine disruptor exposure on participant outcomes, which may introduce bias into the analysis if endocrine disruptor exposure is associated with PM_2.5_ exposure and thyroid hormone levels.

## 5. Conclusions

Our findings suggest that exposure to PM_2.5_ is associated with higher thyroid hormone levels during critical stages of fetal brain development among a susceptible and highly exposed population of pregnant individuals in Puerto Rico. Further research on the relationships between prenatal exposure to air pollution, including fine and ultrafine air pollutants, and adverse neurodevelopmental outcomes in this study population is warranted. Additional research on the exposure sources and mechanisms of action is also necessary to better understand how to intervene and reduce the effects of air pollution on fetal brain development.

## Figures and Tables

**Table 1:** Demographic characteristics of participants in the PROTECT study.

Characteristic	N = 1040 ^1^
**Individual**	
Mother’s age (years)	26.9 ± 5.5
**Socioeconomic**	
Employed at first study visit	
No	390 (37.7%)
Family income per year, USD	
<30,000	587 (64.1%)
Mother’s education	
Less than high school	230 (22.2%)
High school or more	680 (77.8%)
**Medical and Behavioral**	
Number of pregnancies, including this one	
1	450 (43.3%)
2 or more	589 (56.7%)
Number of other children, excluding this one	
0	374 (42.2%)
1 or more	513 (57.9%)
Has experienced adverse birth outcome (spontaneous abortion, preterm birth, or stillbirth)	240 (28.1%)
Exercise habits	
Not exercised for >30 min/day in past 3 months	834 (80.3%)
Maternal pre-pregnancy BMI	
Overweight (25–30)	313 (32.3%)
Obese (>30)	224 (23.1%)
Alcohol consumption	
Any alcohol consumed before pregnancy	427 (41.3%)
Any alcohol consumed during pregnancy	66 (6.4%)
Any cigarette smoke exposure in the home	
Smoke exposure	124 (12.7%)
Marital status at first study visit	
Married	548 (52.9%)
Cohabiting	275 (26.5%)
Single or divorced	211 (20.4%)
^1^ Mean ± SD or n (%)	
Excludes missing values	

## Data Availability

Although analysis code is available, data access is restricted due to participant confidentiality.

## Supplementary Materials

The following supporting information can be downloaded at: https://www.mdpi.com/article/10.3390/toxics13010058/s1, Figure S1: Partially adjusted model sensitivity; Table S1: Data missingness.
