# Supplementary material for: Associations Between Ambient PM2.5 and Thyroid Hormones in Pregnant Persons in Puerto Rico
Source: Toxics. 2025 Jan 15;13(1):58. doi: 10.3390/toxics13010058 (PMC11769198; doi:10.3390/toxics13010058)
Supplement: Supplementary file 1 [file toxics-13-00058-s001.zip › toxics-3389761-supplementary.pdf]

toxics MDPI

Associations Between Ambient $PM_{2.5}$ and Thyroid Hormones in Pregnant Persons in Puerto Rico

Trenton Honda [1,*], Trenton D. Henry [1], Laura Corlin [2,3], Kipruto Kirwa [4], Akram Alshawabkeh [5], Julia R. Varshavsky [5,6], Winston Kennedy [1], José F. Cordero [7], Carmen M. Velez Vega [8], Zaira Y. Rosario Pabon [8], John D. Meeker [9] and Helen Suh [3]

Table S1. Data Missingness.

Variable	Missing*
Visit 1 $PM_{2.5}$	29 (3.3%)
Visit 3 $PM_{2.5}$	16 (2.6%)
Visit 1 TSH	5 (0.6%)
Visit 3 TSH	3 (0.5%)
Visit 1 T3	1 (0.1%)
Visit 3 T3	1 (0.1%)
Visit 1 T4	1 (0.1%)
Visit 3 T4	0 (0%)
Visit 1 FT4_2	0 (0%)
Visit 1 FT4_2	0 (0%)
Visit 1 Month (seasonality)	4 (0.5%)
Visit 3 Month (seasonality)	2 (0.3%)
Race	106 (7.6%)
Mother's education	29 (3.3%)
Mother's age (years)	0 (0%)
Marital status at first study visit	4 (0.4%)
Number of pregnancies, including this one	1 (0.1%)
Number of other children, excluding this one	153 (14.7%)
Employed at first study visit	6 (0.6%)
Household income per year, US dollars	124 (11.9%)
Exercise habits	2 (0.2%)
Maternal pre-pregnancy BMI	72 (6.9%)
Alcohol consumption	5 (0.5%)
Any cigarette smoke exposure in the home	60 (5.8%)

* For specific visits, denominator comprises the subset of cohort that contributed blood samples during the visit; for all other covariates, denominator is the full set of participants (n = 1,040).

Figure S1. Partially Adjusted Model Sensitivity.

Sensitivity analysis of non-overlapping covariates with least missingness.

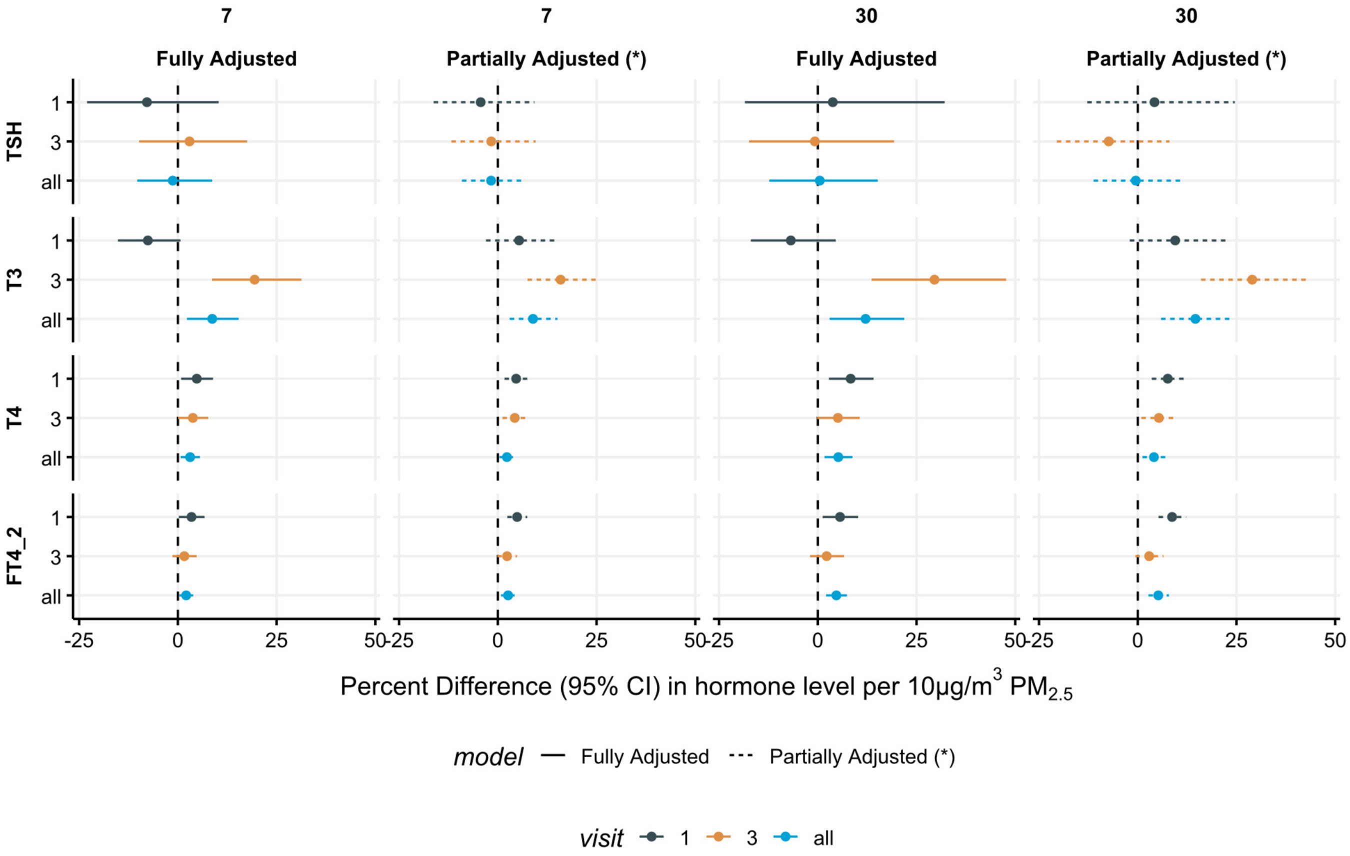

(*) Partially adjusted models include age, smoke exposure, employment, exercise, seasonality (month), and batch.
